# Reply to “Sample Size Calculation. Comment on Quantitative Ultrasound and Dual X-Ray Absorptiometry as Indicators of Bone Mineral Density in Young Women and Nutritional Factors Affecting It, *Nutrients*, 2019, *11*, 2336”

**DOI:** 10.3390/nu12010021

**Published:** 2019-12-20

**Authors:** Katie Schraders, Giancarla Zatta, Marlena Kruger, Jane Coad, Janet Weber, Louise Brough, Jasmine Thomson

**Affiliations:** 1 School of Food and Advanced Technology, College of Sciences, Massey University, Palmerston North 4442, New Zealand; k.schraders@massey.ac.nz (K.S.); giancarlazatta@gmail.com (G.Z.); J.Coad@massey.ac.nz (J.C.); J.L.Weber@massey.ac.nz (J.W.); L.Brough@massey.ac.nz (L.B.); 2 School of Health Sciences, College of Health, Massey University, Palmerston North 4442, New Zealand; M.C.Kruger@massey.ac.nz; 3 Fonterra Research and Development Centre, Fonterra Co-operative Group Limited, Palmerston North 4442, New Zealand

We would like to thank Moran and Sanchez Fernandez [1] for their interest in our work and their comments on our recent publication [2]. We would like to address their concerns regarding our calculation of sample size. We incorrectly described our determination of the sample size and would like to correct our description.

Initially, we determined the sample size for a correlation between DXA and QUS [3] using a type 1 error rate at 5% and type 2 error at 80% with an expected correlation coefficient of at least 0.5 (a moderate effect size), which has been found in other studies. This suggested a sample size of 29 participants. However, we wanted to ensure that we had a representative population including those with low bone mineral density [4]. Thus, the sample size was based on the predicted population of 18–25-year-old females (267,100 in New Zealand in 2013) [5], with an estimated 10% of women of that age having low bone mineral density (10% variability). Assuming a 95% confidence level and 10% precision (margin of error), the minimum sample size needed was 35 women. Factoring in for incomplete data sets/drop-outs between visits of 30%, a sample size of 50 women was required to ensure that 10% were classified as having low bone mineral density.

We hope this letter clarifies the points raised by Moran and Sanchez. We appreciate the opportunity to provide a corrected sample size calculation.
